# Supplementary material for: Resolving the full spectrum of human genome variation using Linked-Reads
Source: Genome Res. 2019 Apr;29(4):635–45. doi: 10.1101/gr.234443.118 (PMC6442396; doi:10.1101/gr.234443.118)
Supplement: Supplemental Material [file supp_gr.234443.118_Supplemental_longranger-2.2.zip › longranger-2.2.tar.bz2!/lr2.2-public/lib/stan/cmdstan/stan/doc/api/html/model__header_8hpp_source.html]

Stan: src/stan/model/model\_header.hpp Source File


|  |  |
| --- | --- |
|  | Stan  2.5.0  probability, sampling & optimization |


- Main Page
- Related Pages
- Namespaces
- Classes
- Files

- File List
- File Members

All Classes Namespaces Files Functions Variables Typedefs Enumerations Enumerator Friends Macros Pages

- src
- stan
- model

model\_header.hpp

Go to the documentation of this file.

1 #ifndef STAN\_\_MODEL\_\_MODEL\_\_HEADER\_HPP\_\_

2 #define STAN\_\_MODEL\_\_MODEL\_\_HEADER\_HPP\_\_

3

4 #include <cmath>

5 #include <cstddef>

6 #include <fstream>

7 #include <iostream>

8 #include <sstream>

9 #include <stdexcept>

10 #include <utility>

11 #include <vector>

12

13 #include <boost/exception/all.hpp>

14 #include <boost/random/linear\_congruential.hpp>

15

16 #include <stan/agrad/rev.hpp>

17 #include <stan/agrad/rev/matrix.hpp>

18

19 #include <stan/agrad/fwd.hpp>

20 #include <stan/agrad/fwd/matrix.hpp>

21

22 #include <stan/io/cmd\_line.hpp>

23 #include <stan/io/dump.hpp>

24 #include <stan/io/reader.hpp>

25 #include <stan/io/writer.hpp>

26 #include <stan/io/csv\_writer.hpp>

27

28 #include <stan/math/matrix.hpp>

29 #include <stan/math.hpp>

30

31 #include <stan/math/rep\_array.hpp>

32 #include <stan/math/rep\_vector.hpp>

33 #include <stan/math/rep\_row\_vector.hpp>

34 #include <stan/math/rep\_matrix.hpp>

35

36 #include <stan/model/prob\_grad.hpp>

37 #include <stan/prob/distributions.hpp>

38

39 #include <stan/common/command.hpp>

40

41 #endif

matrix.hpp

distributions.hpp

matrix.hpp

fwd.hpp

cmd\_line.hpp

rev.hpp

math.hpp

rep\_vector.hpp

command.hpp

rep\_array.hpp

reader.hpp

dump.hpp

rep\_row\_vector.hpp

writer.hpp

csv\_writer.hpp

prob\_grad.hpp

rep\_matrix.hpp

matrix.hpp


---

[ Stan Home Page ]

*© 2011–2014,
Stan Development Team.*
